# A facile synthesis and fungicidal activities of 2-(alkylamino)-5,6-dimethylthieno[2,3-*d*]pyrimidin-4(3*H*)-ones

**DOI:** 10.3762/bjoc.4.49

**Published:** 2008-12-08

**Authors:** Yang-Gen Hu, Ai-Hua Zheng, Xu-Zhi Ruan, Ming-Wu Ding

**Affiliations:** 1 Department of Medicinal Chemistry, Yunyang Medical College, Shiyan 442000, China; 2 Institute of Life Science, Affiliated Taihe Hospital, Yunyang Medical College, Shiyan 442000, China; 3 Key Laboratory of Pesticide & Chemical Biology of Ministry of Education, Central China Normal University, Wuhan 430079, China

**Keywords:** 2-(alkylamino)-5,6-dimethylthieno[2,3-d]pyrimidin-4(3H)-one, aza-Wittig reaction, fungicidal activity, synthesis

## Abstract

The aza-Wittig reactions of iminophosphorane **3** with aromatic isocyanates generated carbodiimides **4**, which were reacted with alkylamines under mild conditions to give a series of 2-(alkylamino)-5,6-dimethylthieno[2,3-*d*]pyrimidin-4(3*H*)-ones **6** and **8** in satisfactory yield. Their structures were confirmed by ^1^H NMR, EI-MS, IR and elementary analysis, and compound **8c** was further analyzed by single-crystal X-ray diffraction. The preliminary bioassays indicated that these compounds showed excellent fungicidal activities against six kinds of fungi.

## Introduction

Over the past ten years, aza-Wittig reactions of functionalized iminophosphoranes with isocyanates have been applied to produce carbodiimides, functional groups consisting of the formula N=C=N, able to undergo a plethora of heterocyclization reactions [1–6]. At the same time, many heterocycles containing thienopyrimidine system are of great importance for use as potential drugs because of their remarkable biological activity. For example, some 2-alkylthio- or 2-alkyl-substituted thienopyrimidinones show significant antifungal and antibacterial activities [7–8], whereas others exhibit good anticonvulsant and angiotensin or H_1_ receptor antagonistic activities [9]. The chemistry of thienopyrimidinones has also received attention because their starting materials, 2-amino-3-carboxythiophenes, can be conveniently synthesized by Gewald reaction [10]. Synthetically useful approaches to thienopyrimidinones starting from easily accessible 2-amino-3-carboxythiophenes are therefore of great importance. Recently we have been interested in the synthesis of a series of new heterocyclic compounds *via* aza-Wittig reaction of α- or β-(ethoxycarbonyl)-substituted iminophosphoranes with aromatic isocyanates and subsequent reaction with various nucleophiles under mild conditions [11–14]. Herein we wish to report an efficient synthesis of 2-(alkylamino)-5,6-dimethylthieno[2,3-*d*]pyrimidin-4(3*H*)-ones via iminophosphorane **3**. Bioassays indicated that these compounds showed good to excellent fungicidal activities against six kinds of fungi.

## Results and Discussion

### Synthesis

**Scheme 1:**
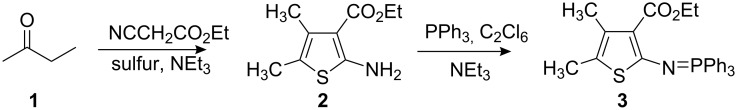
Preparation of iminophosphorane **3**.

The ethyl 2-amino-4,5-dimethylthiophene-3-carboxylate (**2**), easily obtained by cyclization of 2-butanone (**1**) with ethyl 2-cyanoacetate and sulfur under basic conditions [10], was converted to iminophosphorane **3** via reaction with triphenylphosphine, hexachloroethane and triethylamine (Scheme 1).

**Scheme 2:**
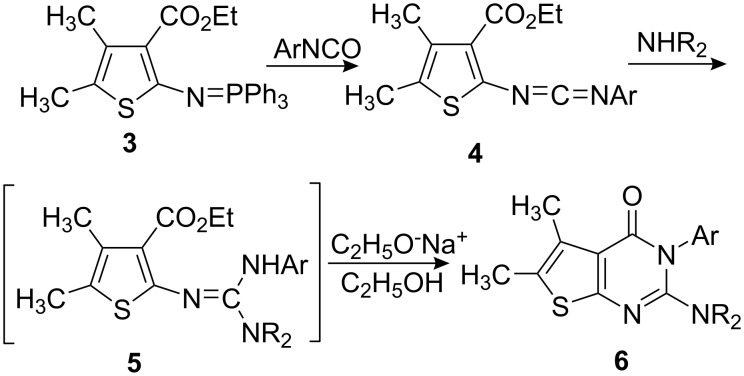
Preparation of 2-(dialkylamino)-5,6-dimethylthieno[2,3-*d*]pyrimidin-4(3*H*)-ones **6**.

Iminophosphorane **3** reacted with aromatic isocyanates to give carbodiimides **4**, which were allowed to react with secondary amines to provide guanidine intermediates **5**. Even in refluxing toluene, compounds **5** did not cyclize. However, in the presence of a catalytic amount of sodium ethoxide, compounds **5** were converted easily to 2-(dialkylamino)-5,6-dimethylthieno[2,3-*d*]pyrimidin-4(3*H*)-ones **6** in satisfactory yields at room temperature (Scheme 2). The results are listed in Table 1.

**Table 1:** Preparation of 2-(alkylamino)-5,6-dimethylthieno[2,3-*d*]pyrimidin-4(3*H*)-ones.

Entry	Product	Ar	−NR_2_ (or −NHR)	Conditions	Yield (%)^a^

1	**6a**	Ph	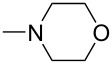	r.t./4 h	76
2	**6b**	Ph	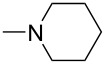	r.t./4 h	73
3	**6c**	Ph	–N(*n*-C_4_H_9_)_2_	r.t./6 h	70
4	**6d**	Ph	–N(*n*-C_6_H_13_)_2_	r.t./6 h	61
5	**6e**	Ph	–N(Me)Ph	r.t./4 h	74
6	**6f**	4-Me-C_6_H_4_	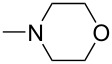	r.t./4 h	78
7	**6g**	4-Cl-C_6_H_4_	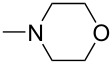	r.t./5 h	86
8	**8a**	Ph	–NH(*t*-C_4_H_9_)	r.t./6 h	71
9	**8b**	3-Me-Ph	–NH(*n*-C_4_H_9_)	r.t./5 h	77
10	**8c**	Ph	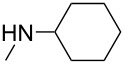	r.t./5 h	75
11	**8d**	Ph	–NH(*n*-C_3_H_7_)	r.t./4 h	73
12	**8e**	4-Cl-C_6_H_4_	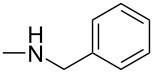	r.t./6 h	71

^a^Yields of isolated products based on iminophosphorane **3**.

**Scheme 3:**
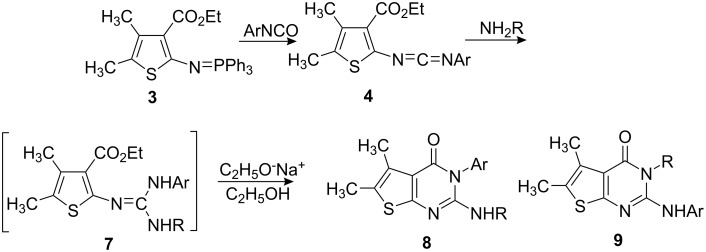
Preparation of 2-alkylamino-5,6-dimethylthieno[2,3-*d*]pyrimidin-4(3*H*)-ones **8**.

The reaction of carbodiimides **4** with primary amine RNH_2_ in the presence of EtONa provided only 2-(alkylamino)-5,6-dimethylthieno[2,3-*d*]pyrimidin-4(3*H*)-ones **8** (Scheme 3), one of the possible regioisomers. We obtained only **8** from the reaction mixture after recrystallization; the other isomer **9** was not found by ^1^H NMR analysis of the reaction mixture.

The structure of **8** was deduced from its ^1^H NMR data. For example, the ^1^H NMR spectrum in **8b** (R = *n*-C_4_H_9_) shows the signals of NH at 4.01 ppm as a broad absorption and NCH_2_ at 3.38–3.31 ppm as a multiple absorption, which strongly suggests the existence of a NHCH_2_CH_2_CH_2_CH_3_ group in **8b**. Furthermore a single crystal of **8c** was obtained from a methylene dichloride solution of **8c** and X-ray structure analysis verified again the proposed structure [15]. The solitary formation of **8** can be rationalized in terms of a base catalyzed cyclization of the guanidine intermediate **7** to give **8** across the arylamino group rather than the alkylamino one. This may probably be due to the preferential generation of –N^−^Ar from more acidic –NHAr. The results are also listed in Table 1.

### Fungicidal activity

The fungicidal activities of compounds **6** and **8** were screened against six kinds of fungi, *Fusarium oxysporum*, *Rhizoctonia solani*, *Botrytis cinerea*, *Gibberella zeae*, *Dothiorella gregaria*, *Colletotrichum gossypii* at a concentration of 50 mg/L according to the reported method [16]. Bioassays indicated that these compounds showed good to excellent fungicidal activities against six kinds of fungi. For example, **6b**, **6d**, **8a**, **8c**, **8d** showed 100% inhibition of *Botrytis cinerea*. See Table 2.

**Table 2:** The fungicidal activities of compounds **6** and **8** (50 mg/L).

Compounds	Relative inhibition (%)					
	F*usarium oxysporum*	*Rhizoctonia solani*	*Botrytis cinerea*	*Gibberella zeae*	*Dothiorella gregaria*	*Colletotrichum gossypii*

**6a**	56	92	97	61	78	84
**6b**	74	99	100	86	93	88
**6c**	63	94	97	67	85	80
**6d**	74	96	100	69	81	80
**6e**	56	98	95	72	85	85
**6f**	70	93	98	64	81	84
**6g**	67	93	99	64	78	86
**8a**	70	87	100	82	78	80
**8b**	74	96	97	65	85	76
**8c**	52	98	100	68	81	81
**8d**	66	93	100	82	93	72
**8e**	71	81	74	44	67	68

In conclusion, we have developed an efficient synthesis of 2-(alkylamino)-5,6-dimethylthieno[2,3-*d*]pyrimidin-4(3*H*)-ones via base-catalyzed reaction of functionalized carbodiimides with various amines. Due to the mild reaction conditions, good yields, easily accessible starting material and straightforward product isolation, we think that the versatile synthetic approach discussed here in many cases compares favorably with other existing methods. The preliminary bioassay of the compounds indicated that the 2-amino-5,6-dimethylthieno[2,3-*d*]pyrimidin-4(3*H*)-ones can be used as lead structure for developing novel fungicides. Further bioassay, optimization and structure-activity relationships of the title compounds are underway.

## Experimental

Melting points were uncorrected. MS were measured on a Finnigan Trace MS spectrometer. IR were recorded on a PE-983 infrared spectrometer as KBr pellets with absorption in cm^−1^. ^1^H NMR spectra were recorded in CDCl_3_ on a Varian Mercury 400 spectrometer and resonances are given in ppm (δ) relative to TMS. Elementary analyses were taken on a Perkin-Elmer CHN 2400 elementary analysis instrument.

### Preparation of [(3-ethoxycarbonyl-4,5-dimethylthiophen-2-yl)imino]triphenylphosphorane (**3**)

To a mixture of ethyl 2-amino-4,5-dimethylthiophene-3-carboxylate (**2**) (2.0 g, 10 mmol), PPh_3_ (5.24 g, 20 mmol) and C_2_Cl_6_ (4.74 g, 20 mmol) in dry CH_3_CN (50 mL), was added dropwise NEt_3_ (4.2 mL, 30 mmol) at room temperature. After stirring for 4–5 h, the solvent was removed under reduced pressure and the residue was recrystallized from EtOH to give iminophosphorane **3** as pale yellow crystals (82% yield), mp 131–133 °C; IR (KBr) cm^−1^ 1693 (C=O), 1484, 1196, 1149, 686; ^1^H NMR (CDCl_3_, 400 MHz) δ: 7.86–7.46 (m, 15H, Ph-H), 4.28 (q, *J* = 7.2 Hz, 2H, OCH_2_), 2.39 (s, 3H, CH_3_), 2.35 (s, 3H, CH_3_), 1.11 (t, *J* = 7.2 Hz, 3H, CH_3_); MS (70 eV) *m/z* (%): 459 (M^+^, 30), 444 (23), 277 (86), 183 (100), 77 (59). Anal. Calcd for C_27_H_26_NO_2_PS: C, 70.57; H, 5.70; N, 3.05; found: C, 70.45; H, 5.55; N, 3.19.

### Preparation of 2-(dialkylamino)-5,6-dimethylthieno[2,3-*d*]pyrimidin-4(3*H*)-ones **6**

To a solution of iminophosphorane **3** (0.92 g, 2 mmol) in dry DCM (15 mL) was added the aromatic isocyanate (2 mmol) under nitrogen at room temperature. After the reaction mixture was stirred for 6–12 h at 0–5 °C, the solvent was removed under reduced pressure and ether/petroleum ether (1:2, 20 mL) was added to precipitate triphenylphosphine oxide. After filtration, the solvent was removed to give carbodiimide **4**, which was used directly without further purification.

To a solution of **4** (prepared above) in DCM (15 ml) was added the secondary amine (2 mmol). After the reaction mixture was stirred for 2–4 h, the solvent was removed and anhydrous ethanol (10 ml) with several drops of EtONa in EtOH were added. The mixture was stirred for 6–12 h at room temperature. The solution was condensed and the residue was recrystallized from ethanol to give 2-(dialkylamino)-5,6-dimethylthieno[2,3-*d*]pyrimidin-4(3*H*)-ones **6**.

**2-Morpholino-3-phenyl-5,6-dimethylthieno[2,3-*d*]pyrimidin-4(3*H*)-one (6a).** White crystals (76% yield), mp: 193–194 °C; IR (KBr) cm^−1^ 1694 (C=O), 1535, 1315, 748; ^1^H NMR (CDCl_3_, 400 MHz) δ: 7.52–7.33 (m, 5H, Ph-H), 3.40 (t, *J* = 4.8 Hz, 4H, 2×OCH_2_), 3.08 (t, *J* = 4.8 Hz, 4H, 2×NCH_2_), 2.39 (s, 3H, CH_3_), 2.35 (s, 3H, CH_3_); MS (70 eV) *m/z* (%): 340/341 (M^+^, 81), 309 (7), 284 (31), 254 (55), 152 (100), 90 (36), 76 (86); Anal. calcd for C_18_H_19_N_3_O_2_S: C, 63.32; H, 5.61; N, 12.31; found: C, 63.25, H, 5.69; N, 12.45.

**2-Piperidino-3-phenyl-5,6-dimethylthieno[2,3-*d*]pyrimidin-4(3*H*)-one (6b).** White crystals (73% yield), mp: 140–141 °C; IR (KBr) cm^−1^ 1690 (C=O), 1529, 1319, 745; ^1^H NMR (CDCl_3_, 400 MHz) δ: 7.49–7.31 (m, 5H, Ph-H), 3.05 (t, *J* = 5.6 Hz, 4H, 2×NCH_2_), 2.39 (s, 3H, CH_3_), 2.35 (s, 3H, CH_3_), 1.39–1.18 (m, 6H, 3×CH_2_); MS (70 eV) *m/z* (%): 339 (M^+^, 100), 310 (21), 254 (49), 194 (37), 152 (97), 76 (71); Anal. calcd for C_19_H_21_N_3_OS: C, 67.23; H, 6.24; N, 12.38; found: C, 67.16; H, 6.26; N, 12.42.

**2-(Dibutylamino)-3-phenyl-5,6-dimethylthieno[2,3-*d*]pyrimidin-4(3*H*)-one (6c):** White crystals (70% yield), mp: 87–89 °C; IR (KBr) cm^−1^ 1688 (C=O), 1530, 1320, 742; ^1^H NMR (CDCl_3_, 400 MHz) δ: 7.70–7.26 (m, 5H, Ph-H), 2.97–2.93 (t, *J* = 7.2, 4H, 2×NCH_2_), 2.38 (s, 3H, CH_3_), 2.34 (s, 3H, CH_3_), 1.22–1.09 (m, 8H, 2×CH_2_CH_2_CH_2_), 0.82 (t, *J* = 7.2, 6H, 2×CH_3_); MS (70 eV) *m/z* (%): 383 (M^+^, 100), 354 (6), 340 (15), 326 (28), 281/282 (29), 254/255 (34), 90 (11), 76 (32); Anal. calcd for C_22_H_29_N_3_OS: C, 68.89; H, 7.62; N, 10.96; found: C, 68.76; H, 7.68; N, 10.82.

**2-(Dihexylamino)-3-phenyl-5,6-dimethylthieno[2,3-*d*]pyrimidin-4(3*H*)-one (6d):** White crystals (61% yield), mp: 70–71 °C; IR (KBr) cm^−1^ 1689 (C=O), 1530, 1323, 745; ^1^H NMR (CDCl_3_, 400 MHz) δ: 7.48–7.26 (m, 5H, Ph-H), 2.97–2.93 (t, *J* = 7.2, 4H, 2×NCH_2_), 2.38 (s, 3H, CH_3_), 2.34 (s, 3H, CH_3_), 1.27–1.08 (m, 16H, 2×CH_2_CH_2_CH_2_CH_2_), 0.86 (t, *J* = 7.2, 6H, 2×CH_3_); MS (70 eV) *m/z* (%): 439 (M^+^, 100), 368 (6), 354 (10), 281/282 (10), 77/76 (6); Anal. calcd for C_26_H_37_N_3_OS: C, 71.03; H, 8.48; N, 9.56; found: C, 70.94; H, 8.50; N, 9.60.

**2-(*N*-Methyl-*N*-phenylamino)-3-phenyl-5,6-dimethylthieno[2,3-*d*]pyrimidin-4(3*H*)-one (6e):** White crystals (74% yield), mp: 194–196 °C; IR (KBr) cm^−1^ 1684 (C=O), 1530, 1315, 746; ^1^H NMR (CDCl_3_, 400 MHz) δ: 7.10–6.57 (m, 10H, Ph-H), 3.25 (s, 3H, NCH_3_), 2.40 (s, 3H, CH_3_), 2.38 (s, 3H, CH_3_); MS (70 eV) *m/z* (%): 361/360 (M^+^, 84), 254 (8), 103 (17), 90 (8), 76 (100), 58 (19); Anal. calcd for C_21_H_19_N_3_OS: C, 69.78; H, 5.30; N, 11.62; found: C, 69.70; H, 5.18; N, 11.53.

**2-Morpholino-3-(4-methylphenyl)-5,6-dimethylthieno[2,3-*d*]pyrimidin-4(3*H*)-one (6f):** White crystals (78% yield), mp: 164–166 °C; IR (KBr) cm^−1^ 1685 (C=O), 1524, 1116, 743; ^1^H NMR (CDCl_3_, 400 MHz) δ: 7.30–7.20 (m, 4H, Ph-H), 3.42 (t, *J* = 4.8 Hz, 4H, 2×OCH_2_), 3.09 (t, *J* = 4.8 Hz, 4H, 2×NCH_2_), 2.41 (s, 3H, Ph-CH_3_), 2.39 (s, 3H, CH_3_), 2.35 (s, 3H, CH_3_); MS (70 eV) *m/z* (%): 355 (M^+^, 100), 324 (8), 310 (20), 269 (23), 153 (28), 91 (22); Anal. calcd for C_19_H_21_N_3_O_2_S: C, 64.20; H, 5.95; N, 11.82; found: C, 64.06; H, 5.61; N, 11.75.

**2-Morpholino-3-(4-chlorophenyl)-5,6-dimethylthieno[2,3-*d*]pyrimidin-4(3*H*)-one (6g):** White crystals (86% yield), mp: 173–175 °C; IR (KBr) cm^−1^ 1689 (C=O), 1528, 1320, 741; ^1^H NMR (CDCl_3_, 400 MHz) δ: 7.48–7.28 (m, 4H, Ph-H), 3.44 (t, *J* = 4.8 Hz, 4H, 2×OCH_2_), 3.08 (t, *J* = 4.8 Hz, 4H, 2×NCH_2_), 2.38 (s, 3H, CH_3_), 2.35 (s, 3H, CH_3_); MS (70 eV) *m/z* (%): 375 (M^+^, 100), 340 (54), 289/290 (19), 162 (24), 110 (78), 90 (29), 77/76 (23); Anal. calcd for C_18_H_18_ClN_3_O_2_S: C, 57.52; H, 4.83; N, 11.18; found: C, 57.45; H, 4.76; N, 11.20.

### Preparation of 2-(alkylamino)-5,6-dimethylthieno[2,3-*d*]pyrimidin-4(3*H*)-ones 8

To the solution of **4** (2 mmol) prepared above in DCM (10 ml) was added the primary amine (2 mmol). After the reaction mixture was stirred for 5–6 h, the solvent was removed and anhydrous ethanol (10 ml) with several drops of EtONa in EtOH were added. The mixture was stirred for 6–12 h at room temperature. The solution was condensed and the residue was recrystallized from ethanol to give 2-(alkylamino)-5,6-dimethylthieno[2,3-*d*]pyrimidin-4(3*H*)-ones **8**.

**2-(*tert*-Butylamino)-5,6-dimethyl-3-phenylthieno[2,3-*d*]pyrimidin-4(3*H*)-one (8a).** White crystals (71% yield), mp: 184–186 °C; IR (KBr) cm^−1^ 1692 (C=O), 1526, 1120, 748; ^1^H NMR (CDCl_3_, 400 MHz) δ: 7.56–7.16 (m, 5H, Ph-H), 3.82 (s, 1H, NH), 2.35 (s, 3H, CH_3_), 2.31 (s, 3H, CH_3_), 1.34 (s, 9H, 3×CH_3_); MS (70 eV) *m/z* (%): 327 (M^+^, 93), 271 (91), 255 (31), 153 (100), 119 (19), 77 (23); Anal. calcd for C_18_H_21_N_3_OS: C, 66.02; H, 6.46; N, 12.83; found: C, 66.22; H, 6.57; N, 12.97.

**2-(Butylamino)-5,6-dimethyl-3-(3-methylphenyl)thieno[2,3-***d*]pyrimidin-4(3***H*)-one (8b).** White crystals (77% yield), mp: 202–204 °C; IR (KBr) cm^−1^ 1697 (C=O), 1543, 1120, 748; ^1^H NMR (CDCl_3_, 400 MHz) δ: 7.48–7.06 (m, 4H, Ph-H), 4.01 (s, 1H, NH), 3.38–3.31 (m, 2H, NCH_2_), 2.42 (s, 3H, CH_3_), 2.37 (s, 3H, CH_3_), 2.32 (s, 3H, CH_3_), 1.49–1.22 (m, 4H, 2×CH_2_), 0.88 (t, *J* = 7.2, 3H, CH_3_); MS (70 eV) *m/z* (%): 341.2 (M^+^, 95), 285 (60), 153 (100), 133 (18), 105 (26), 91 (39); Anal. calcd for C_19_H_23_N_3_OS: C, 66.83; H, 6.79; N, 12.31; found: C, 66.79; H, 6.88; N, 12.42.

**2-(Cyclohexylamino)-5,6-dimethyl-3-phenylthieno[2,3-*d*]pyrimidin-4(3*H*)-one (8c).** White crystals (75% yield), mp: 198–200 °C; IR (KBr) cm^−1^ 1698 (C=O), 1540, 1128, 740; ^1^H NMR (CDCl_3_, 400 MHz) δ: 7.48–7.25 (m, 5H, Ph-H), 4.02 (s, 1H, NH), 2.37 (s, 3H, CH_3_), 2.35 (s, 3H, CH_3_), 2.00–1.96 (m, 1H, CH), 1.62–1.60 (m, 4H, 2×CH_2_), 1.44–1.02 (m, 6H, 3×CH_2_); MS (70 eV) *m/z* (%): 353 (M^+^, 44), 270 (34), 153 (100), 133 (24), 98 (19), 91 (52); Anal. calcd for C_20_H_23_N_3_OS: C, 67.96; H, 6.56; N, 11.89; found: C, 68.08; H, 6.71; N, 11.78.

**2-(Propylamino)-5,6-dimethyl-3-phenylthieno[2,3-*d*]pyrimidin-4(3*H*)-one (8d).** White crystals (73% yield), mp: 212–214 °C; IR (KBr) cm^−1^ 1689 (C=O), 1530, 1130, 740; ^1^H NMR (CDCl_3_, 400 MHz) δ: 7.61–7.28 (m, 5H, Ph-H), 4.00 (s, 1H, NH), 3.34–3.29 (m, 2H, NCH_2_), 2.36 (s, 3H, CH_3_), 2.31 (s, 3H, CH_3_), 1.52–1.46 (m, 2H, CH_2_), 0.83 (t, *J* = 7.2, 3H, CH_3_); MS (70 eV) *m/z* (%): 313 (M^+^, 77), 270 (79), 255 (22), 153 (100), 119 (31), 77 (34); Anal. calcd for C_17_H_19_N_3_OS: C, 65.15; H, 6.11; N, 13.41; found: C, 65.28; H, 6.32; N, 13.60.

**2-(Benzylamino)-3-(4-chlorophenyl)-5,6-dimethylthieno[2,3-*d*]pyrimidin-4(3*H*)-one (8e).** White crystals (71% yield), mp: 248–250 °C; IR (KBr) cm^−1^ 1698 (C=O), 1538, 1130, 747; ^1^H NMR (CDCl_3_, 400 MHz) δ: 7.52–7.20 (m, 9H, Ph-H), 4.57 (s, 2H, CH_2_), 4.32 (s, 1H, NH), 2.36 (s, 3H, CH_3_), 2.31 (s, 3H, CH_3_); MS (70 eV) *m/z* (%): 395 (M^+^, 100), 330 (48), 270 (12), 201 (74), 153 (24), 91 (31); Anal. calcd for C_21_H_18_ClN_3_OS: C, 63.71; H, 4.58; N, 10.61; found: C, 63.80; H, 4.72; N, 10.44.

## References

[1] Ulrich H (2007). Chemistry and Technology of Carbodiimides.

[2] Zhao M-X, Wang M-X, Yu C-Y, Huang Z-T, Fleet G W J (2004). J Org Chem.

[3] Csámpai A, Túrós G, Kudar V, Simon K, Oeynhausen H, Wamhoff H, Sohár P (2004). Eur J Org Chem.

[4] Hao J, Xia Y, Wang L, Ruhlmann L, Zhu Y, Li Q, Yin P, Wei Y, Guo H (2008). Angew Chem, Int Ed.

[5] Li Q, Wei Y, Hao J, Zhu Y, Wang L (2007). J Am Chem Soc.

[6] Wei Y, Xu B, Barnes C L, Peng Z (2001). J Am Chem Soc.

[7] Walter H (1999). Novel Pyrimidin-4-one and Pyrimidin-4-thione as Fungicide. PCT Int. Pat. Appl..

[8] Chambhare R V, Khadse B G, Bobde A S, Bahekar R H (2003). Eur J Med Chem.

[9] Shishoo C J, Shirsath V S, Rathod I S, Yande V D (2000). Eur J Med Chem.

[10] Sabnis R W, Rangnekar D W, Sonawane N D (1999). J Heterocycl Chem.

[11] Sun Y, Huang N Y, Ding M W (2009). Synth Commun.

[12] Ding M-W, Xu S-Z, Zhao J-F (2004). J Org Chem.

[13] Yuan J-Z, Fu B-Q, Ding M-W, Yang G-F (2006). Eur J Org Chem.

[14] Zhao J-F, Xie C, Xu S-Z, Ding M-W, Xiao W-J (2006). Org Biomol Chem.

[15] Zheng A, Xu J, Hu Y-G (2006). Acta Crystallogr, Sect E.

[16] Ren Q, Cui Z, He H, Gu Y (2007). J Fluorine Chem.

